# Telomere Shortening in Hematological Malignancies with Tetraploidization—A Mechanism for Chromosomal Instability?

**DOI:** 10.3390/cancers9120165

**Published:** 2017-11-30

**Authors:** Eigil Kjeldsen

**Affiliations:** Cancercytogenetic Section, HemoDiagnostic Laboratory, Department of Hematology, Aarhus University Hospital, Tage-Hansens Gade 2, Ent. 4A, DK-8000 Aarhus C, Denmark, Eigil.Kjeldsen@clin.au.dk; Tel.: +45-7846-7799; Fax: +45-7846-7399

**Keywords:** tetraploidy, telomere length, chromosomal instability, hematological malignancy, iQFISH

## Abstract

Aneuploidy, the presence of an abnormal number of chromosomes in a cell, is one of the most obvious differences between normal and cancer cells. There is, however, debate on how aneuploid cells arise and whether or not they are a cause or a consequence of tumorigenesis. Further, it is important to distinguish aneuploidy (the “state” of the karyotype) from chromosomal instability (CIN; the “rate” of karyotypic change). Although CIN leads to aneuploidy, not all aneuploid cells exhibit CIN. One proposed route to aneuploid cells is through an unstable tetraploid intermediate because tetraploidy promotes chromosomal aberrations and tumorigenesis. Tetraploidy or near-tetraploidy (T/NT) (81–103 chromosomes) karyotypes with or without additional structural abnormalities have been reported in acute leukemia, T-cell and B-cell lymphomas, and solid tumors. In solid tumors it has been shown that tetraploidization can occur in response to loss of telomere protection in the early stages of tumorigenesis in colon cancer, Barrett’s esophagus, and breast and cervical cancers. In hematological malignancies T/NT karyotypes are rare and the role of telomere dysfunction for the induction of tetraploidization is less well characterized. To further our understanding of possible telomere dysfunction as a mechanism for tetrapolydization in hematological cancers we here characterized the chromosomal complement and measured the telomere content by interphase nuclei quantitative fluorescence in situ hybridization (iQFISH) in seven hematological cancer patients with T/NT karyotypes, and after cytogenetic remission. The patients were identified after a search in our local cytogenetic registry in the 5-year period between June 2012 and May 2017 among more than 12,000 analyzed adult patients in this period. One advantage of measuring telomere content by iQFISH is that it is a single-cell analysis so that the telomere content can be distinguished between normal karyotype cells and cells with T/NT karyotypes. We find that the telomeres are particularly short in cells with T/NT karyotypes as compared with normal cells, and in T/NT karyotypes harboring additional chromosomal aberrations as well. These findings suggest that telomere dysfunction in hematological malignancies may be a mechanism for tetraploidization and CIN.

## 1. Introduction

Tetraploidy (4n, 92 chromosomes) without other numerical or structural abnormalities may be observed at a low level (<10% of analyzed cells) in a normal bone marrow (BM) [1]. This type of tetraploidy may represent mitotic megakaryocytes or cells at anaphase stage in which paired sister chromatids are separated. Tetraploid or near-tetraploid (T/NT) (81–103 chromosomes) karyotypes harboring additional numerical and/or structural abnormalities have been associated with tumorigenesis and are often observed in solid tumors [2], and more rarely in hematological malignancies [3].

Three main mechanisms for tetraploidization in cancer have been proposed: cell fusion, a failure to complete mitosis (mitotic slippage), and a failure to complete cytokinesis [4]. In a mouse model system, permanently dysfunctional telomeres have been shown to cause by-pass of mitosis and tetraploidization [5], promoting tumorigenic transformation [6]. In humans, telomere shortening occurs during normal ageing, and critical short telomeres have been reported as a common alteration in many epithelial cancers, the prevalent tumor type in the elderly [7]. Critical short or dysfunctional telomeres in human fibroblasts and mammary epithelial cells can induce tetraploidization [6], which has the potential to promote epithelial carcinogenesis in early cancerous lesions [2,8,9]. In epithelial cancers it has been suggested that tetraploidy is an intermediate of chromosomal instability (CIN) because tetraploid cells tend to lose chromosomes progressively during aberrant bipolar mitoses [10] and/or undergo multipolar mitosis during which chromosomes are distributed among the daughter cells in a near-to-random manner [11,12].

Tetraploidization in hematological malignancies is rare and its role in CIN and tumorigenesis is less well characterized [13]. Here we characterized the chromosomal complement and measured the telomere content in diploid and T/NT cells at diagnosis, and at complete remission (CR), in seven patients with various hematological malignancies.

## 2. Materials and Methods

### 2.1. Patient Cohort

The study is population-based, including adult patients with hematological malignancies referred to our department for cytogenetic diagnostics from the Central Denmark Region and the Nothern Denmark Region. The patients’ bone marrow aspirates were prospectively examined by conventional cytogenetic analysis. Patients were selected by a search in our patient registry in the five-year period from June 2012 to May 2017. The selection criteria were karyotypes with T/NT present in 3 or more metaphases out of 20 to 25 analyzed G-banded metaphases from patients diagnosed with a hematological malignancy. Patients with multiple myeloma were excluded.

Seven cases were identified among more than 10,000 adult patients examined in the study period: 3 cases with de novo acute myeloid leukemia (AML), 1 case with secondary AML after myelodysplastic syndrome (MDS), 1 case with B-cell acute lymphoblastic leukemia (B-ALL), 1 case with intestinal myeloid sarcoma and bone marrow involvement, and 1 case with lymphoma (Table 1), and paired remission samples were identified afterwards. Six patients obtained cytogenetic and hematological remission 1–4 months after diagnosis and chemotherapeutic treatment, while one patient (Case No. 2) deceased 5 weeks after diagnosis before cytogenetic remission could be evaluated.

### 2.2. G-Banding Karyotyping

Conventional cytogenetic analysis (CCA) by G-banding was done as described previously [14]. Briefly, 24-h un-stimulated bone marrow cell cultures were established in RPMI-1640 medium supplemented with 20% fetal bovine serum. Harvest, fixation, and G-banding were done according to standard procedures, and 25 metaphases were analyzed using a charged coupled device (CCD)-based imaging system (Ikaros, MetaSystems, Altlussheim, Germany). Karyotypes were described according to the International System for Human Cytogenetic Nomenclature (ISCN), (2013) [15]. All residual cell suspensions used for cytogenetic examination were stored at −20 °C.

### 2.3. Twenty-Four Hour Color Karyotyping

Analysis by 24-color karyotyping was done using the 24XCyte kit according to manufacturer’s instructions (MetaSystems, Altlussheim, Germany).

### 2.4. Fluorescence In Situ Hybridization (FISH)

All FISH analyses were performed on the same cell suspension as used for CCA. Directly labelled arms-specific painting probes (Leica Biosystems, Nussloch, Germany) representing chromosome arms 1p, 1q, 2p and 2q were used according to manufacturer’s instructions. Directly-labelled locus specific probes for the loci of *TP53*, *RB1* and *CBFB* (MetaSystems, Altlussheim, Germany). FISH analyses were done according to manufacturer’s instructions. Metaphase chromosomes and interphase nuclei were counterstained with DAPI (4’,6-diamidino-2-phenylindole)/antifade solution.

### 2.5. Quantification of Telomere Content in Diploid and Tetraploid Cells by FISH

Telomere measurement was done in one case (Case 2) by metaphase telomere/centromere-FISH (T/C-FISH) at the time of diagnosis as a request of the referring clinicians. Retrospectively, the telomere content was measured in all cases by interphase nuclei quantitative FISH (iQ-FISH) on the same bone marrow cell suspension as previously used for CCA in samples from the time of diagnosis and at the time of obtained cytogenetic remission. Bone marrow cell suspension from a normal age-matched male served as control.

For T/C-FISH, metaphase preparation, hybridization and detection were done according to manufacturer’s instructions using the Telolomere FISH kit (Agilent/DAKO Denmark A/S, Glostrup, Denmark). Briefly, the kit contains a pan-telomeric (G_3_TA_2_) FITC-(fluorescein isothiocyanate) labelled PNA (peptide nucleic acid) probe and as internal control a FITC-labelled PNA probe for the centromere of chromosome 2. After hybridization and washing steps metaphases were counterstained with DAPI/antifade solution before slide scanning using the automated fluorescence-based microscope scanning system, Metafer4 (MetaSystems, Altlussheim, Germany). For telomere measurement the chromosomes of each metaphase were arranged in to a karyogram and the ISIS-Telomere module (MetaSystems) was applied as described in [16]. The software calculates a T/C value that has been shown to be in a tight linear relationship with classical telomere restriction fragment (TRF) of different individuals by Southern blot. To determine TRF values from T/C-FISH data simple regression analysis of medians was applied thus representing the telomere length of each individual chromosome arm or as an average from all of the chromosomes in one metaphase [16,17]. Ten metaphases were evaluated and T/C values from diploid cells and T/NT cells were scored and recorded.

iQ-FISH was performed using the pan-telomeric (G_3_TA_2_) Cy3-labeled PNA probe and as internal reference, a chromosome 2 centromere-specific FITC-labelled PNA probe (Agilent/DAKO) as described in [18,19,20]. Briefly, nuclei were counterstained with DAPI before slide scanning, intensity measurements, and quantification using the automated Metafer4 system (MetaSystems). The MetaCyte software included in the scanning system allows for automated capture as well as image and processing steps. The ratio between the Cy3 and FITC intensities multiplied by 100 were automatically calculated and recorded as fluorescence ratio units (FRU) representing the relative telomere content. A minimum of 250 nuclei was scanned for every sample. Scanned nuclei were visually evaluated to exclude possible artifacts from the scanning result, such as unfocussed nuclei or images with more than one nucleus.

To compare the telomere content in tetraploid and diploid nuclei at diagnosis and at CR, each nucleus was scored with respect to the number of green centromere 2 signals it contained, where diploid cells contained two green signals and tetraploid cells contained four green signals. The corresponding FRU values were recorded.

### 2.6. Measurement of Cell Size in Diploid and T/NT Cells

In addition, the MetaCyte software automatically measures and records the contour area (µm^2^) representing the size of each scanned nucleus. To compare the cell size in tetraploid and diploid nuclei at diagnosis, and at CR, each nucleus was scored with respect to the number of green centromere 2 signals it contained, as described above, and the corresponding contour areas were recorded.

### 2.7. Statistical Analysis

Statistical analyses were performed using GraphPad Prism software version 6.0 h (GraphPad Software Inc., La Jolla, CA, USA). Unpaired t-testing was performed to evaluate the significance of variant telomere content and contour area between diploid and tetraploid cells at diagnosis and at CR. Analysis of variance (ANOVA) was tested for in each group and if the hypothesis of homogeneity of variance was rejected Welch’s test was applied [21]. Values of *p* < 0.05 were considered as statistically significant.

## 3. Results and Discussion

**Figure 1:**
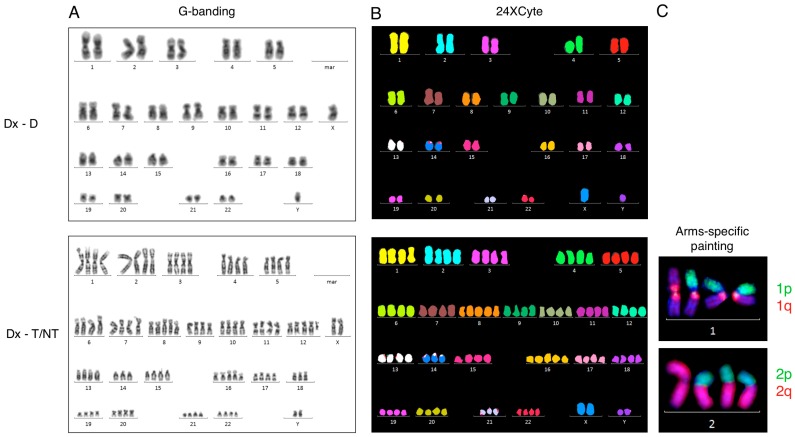
G-banding, 24-color karyotyping, and arms-specific chromosome painting. (**A**) G-banded karyotype at diagnosis showing a normal diploid karyotype (upper panel, Dx-D) and a near-tetraploid karyotype (lower panel, Dx-T/NT). (**B**) 24-color karyotyping at diagnosis showing a normal diploid karyotype (upper panel) and a near-tetraploid karyotype (lower panel). (**C**) A partial karyotype of near-tetraploid cells after arms-specific chromosome painting with 1p and 1q probes (upper panel) and with 2p and 2q (lower panel). Dx-D is diploid scored cells at diagnosis, and Dx-T/NT is tetraploid/near-tetraploid scored cells at diagnosis.

Initially, we examined an 82-year-old male with intestinal sarcoma and AML affecting his bone marrow as part of a routine cytogenetic diagnostic work-up. A near-tetraploid (T/NT) karyotype with 91 to 94 chromosomes and additional structural aberrations were recognized in 12 out 25 analyzed metaphases (Figure 1A). The T/NT karyotype was confirmed by 24-color karyotyping (Figure 1B). Chromosome painting with arms-specific probes for chromosomes 1p, 1q, 2p, and 2q confirmed the presence of isochromosomes for chromosomes 1 and 2 in the T/NT cells as well (Figure 1C). A complete karyotype of this patient is given in Table 1 (Case 2).

**Figure 2:**
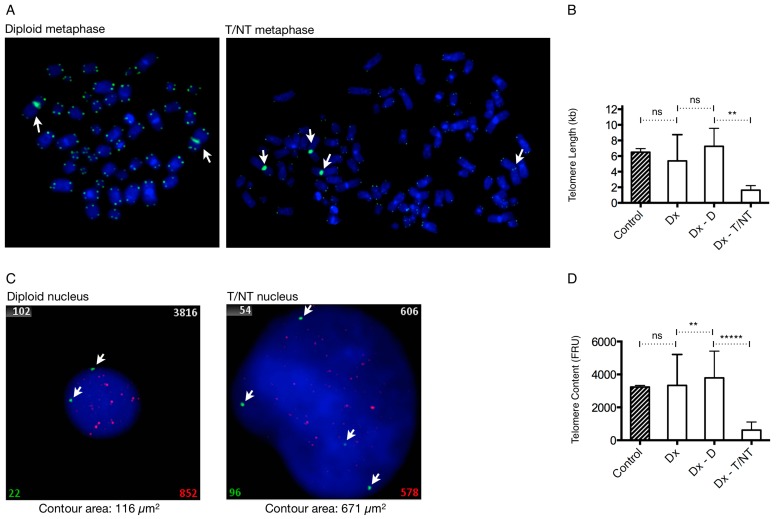
Telomere quantification with telomere/centromere fluorescence in situ hybridization (T/C FISH). (**A**) Representative metaphases from a diploid metaphase (left-hand panel) and a near-tetraploid metaphase (right-hand panel). Telomere signals have a lower intensity in the near-tetraploid metaphase than in the diploid metaphase or are even missing in some chromosome arms. (**B**) Mean telomere lengths (kilobases) in a normal healthy age-matched control and in Case 2 at diagnosis, where telomere measurements are from un-sorted metaphases at (Dx) or after ploidy scoring into diploid metaphases (Dx-D) and near-tetraploid metaphases (Dx-T/NT). Near-tetraploid metaphases show significantly shorter telomeres compared with diploid metaphases (*p* = 0.0013), whereas there were no significant differences between the other groups. (**C**) Representative nuclei with diploid chromosomal complement (left-hand panel) and T/NT chromosomal complement (right-hand panel) are shown together with their respective contour area in µm^2^. The fluorescence intensity of chromosome 2 centromeric and pan-telomeric probes is displayed in the left and right bottom corners, respectively. The ratio between telomeric and centromeric 2 fluorescence intensities multiplied by 100 is displayed in the upper right corner. Cell number is displayed in the upper left corner. (**D**) Mean telomere content defined as FRU (fluorescence ratio units) determined in the same healthy age-matched control as before and in Case 2 at diagnosis, where telomere measurements are from un-sorted nuclei (Dx) or after ploidy scoring into diploid nuclei (Dx-D) and near-tetraploid nuclei (Dx-T/NT). Near-tetraploid nuclei have significantly lower telomere content compared with diploid cells (*p* < 0.0001). There was no significant difference between un-sorted nuclei and nuclei from the control. White arrows indicate centromere 2 signals. After significance testing number of asterisk indicate significance level: ****: extremely significant (*p* < 0.0001); ***: extremely significant (0.0001 < *p* > 0.001); **: very significant (0.001 < *p* > 0.01); *: significant (0.01 < *p* > 0.05); and ns: not significant (*p* ≥ 0.05). Dx is unsorted cells at diagnosis, Dx-D is scored diploid cells at diagnosis, and Dx-T/NT is tetraploid/near-tetraploid scored cells at diagnosis.

In addition, at the time of diagnosis telomere length analysis was also requested. At that time, we performed average telomere length measurements using the T/C metaphase FISH method [16,17]. After hybridization with FITC-labeled PNA probes for telomeres and centromere 2 we analyzed metaphases by the ISIS-Telomere module. The software calculates a T/C value, a measure for telomere length for each individual chromosome, which can be converted to an average telomere length for each analyzed metaphase [16]. We identified seven diploid metaphases and four T/NT metaphases, which could be analyzed. The average T/C values for each metaphase were calculated and grouped according to ploidy generating a mean T/C value representing the average telomere length in diploid and T/NT metaphases. We observed a significant reduction in average telomere length in the T/NT metaphases compared with the telomere length in diploid metaphases (*p* = 0.0013) (Figure 2A,B). Further, we found that when the analyzed metaphases were not grouped according to ploidy there was no significant difference in average T/C values when compared with a normal age-matched control (Figure 2B). Thus, by analyzing the average T/C values in diploid and T/NT metaphases we found that the average telomere length was reduced by a factor of 4.4 compared with diploid metaphases. Data from previous studies in mice and human fibroblasts suggested that reduced telomere length plays an important role in the induction of tetraploidy [5,6], which is in support of our present findings.

The T/C metaphase FISH telomere method requires that cell types to be analyzed are mitotically active, and the chromosomes in the metaphase should not cross each other and be rather straight in order for the metaphases to be amenable for analysis [16]. Furthermore, it is very labor-intensive to generate a sufficient number of karyograms for measurements and there may be a selection bias with respect to the cell types that are mitotically active in the sample. These types of problems can be overcome by quantitative fluorescence in situ hybridization of interphase nuclei (iQFISH) measuring relative telomere content in interphase nuclei [22]. We have previously utilized this T/C iQFISH method to investigate telomeres in a patient’s bone marrow cells with or without genomic aberrations in the same sample [20].

We applied the T/C iQFISH method in our AML patient with tetraploidy. The bone marrow sample of Case 2 and the age-matched control were hybridized with a Cy3-labeled PNA telomere probe and an FITC-labeled PNA centromere 2 probe. Images of at least 250 nuclei in each case were captured automatically and analyzed by the ISIS-Telomere module [19,22]. The software calculates a relative telomere to centromere (T/C) fluorescence ratio unit (FRU) multiplied by 100 [19,20]. The relative telomere content determined as FRU eliminates variations due to uneven hybridization efficiency [19]. The captured images of the nuclei were then scored manually according to ploidy as determined by the number of centromere 2 signals each nucleus contained, where two signals represent diploid nuclei and four signals represent T/NT nuclei. The respective FRU values were grouped with respect to ploidy and mean FRU values were calculated in the different groups (Figure 2C,D). The T/C iQFISH method showed that T/NT nuclei had a significantly reduced telomere content compared with diploid nuclei (*p* < 0.0001). The telomere content in the T/NT cells was reduced by a factor of 6.2, which is in line with the T/C metaphase FISH result. Furthermore, there was no significant difference in telomere content when nuclei were not grouped according to ploidy and the age-matched control. Together, these data suggest that it is important to distinguish between diploid and T/NT nuclei to detect possible differences in telomere content between subtypes of ploidy especially if T/NT cells are present in only a small fraction.

Another apparent difference between T/NT and diploid nuclei is the size of the examined nuclei (Figure 2C). The ISIS-Telomere software has the advantage that it automatically calculates the contour area of each analyzed cell in addition to the telomere measurements described above. This means that it is possible from the same hybridization experiment to determine both the telomere content and contour area of each analyzed cell in which the contour area is equivalent to cell size. Measurement of the contour area in nuclei with T/NT and diploid chromosomal complement, as distinguished by the respective numbers of green centromere 2 signals they contained, showed that the contour area of T/NT nuclei (671 µm^2^) were considerably enlarged compared with diploid cells (116 µm^2^). This finding is in agreement with previous studies showing that increased DNA content is directly related to blast size [23].

To examine whether it is a general phenomenon that T/NT cells have reduced telomere length and enlarged nucleus size, and how this may be associated with additional chromosomal aberrations, we performed a search in our local cytogenetic registry for additional patients with T/NT karyotypes. Six additional patients were identified in the period between 2012 and 2017 (Table 1). Five of these seven patients had AML, one had follicular lymphoma (FL), and one had B-cell acute lymphoblastic leukemia (B-ALL). We also wanted to compare the diagnostic sample with paired samples obtained at complete remission.

**Figure 3:**
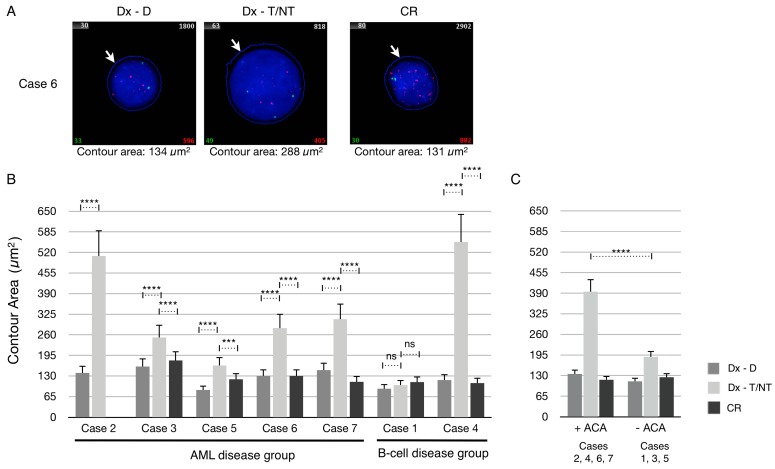
Mean nucleus size in the AML and B-cell disease groups. (**A**) Representative nuclei from Case 6 at diagnosis after ploidy scoring and at complete remission (CR). The contour area (µm^2^) of representative nuclei is indicated below each image. White arrows indicate nucleus boundary determined automatically according to DAPI stain. (**B**) Mean contour area of cases determined at diagnosis and after ploidy scoring being divided into diploid (Dx-D) or as T/NT (Dx-T/NT), and at CR. At CR there were no clonal T/NT cells present in either of the samples. (**C**) Average contour area calculated from contour area of all nuclei when grouped according to presence of additional chromosomal aberrations (+ACA) or their absence (−ACA) after ploidy scoring into diploid (Dx-D) or T/NT (Dx-T/NT) nuclei, and at CR.

For these purposes each patient sample was first hybridized with a Cy3-labeled PNA telomere probe and FITC-labeled PNA centromere 2 probe, and automatically analyzed by the ISIS-Telomere software. First, we examined the dataset of the contour area measurements from each patient after manual ploidy scoring of analyzed cells. The contour area of the nuclei at diagnosis (Dx-D and Dx–T/NT) and at CR were determined by the ISIS-Telomere software, which utilizes the DAPI counterstain to indicate the nucleus boundary as illustrated in Figure 3A. The mean contour area at diagnosis (Dx-D and Dx–T/NT) and at CR of all included cases was determined and plotted (Figure 3B). Comparing the mean contour area of T/NT cells from each patient at diagnosis showed a great variability between these. For example, in Case 2 and Case 4 the mean contour area of the T/NT cells is enlarged by approximately 4 times compared with diploid cells, whereas in Case 1 there was no difference in contour area between diploid and T/NT nuclei. It can also be seen that the contour area at CR in all cases is similar to diploid cells at diagnosis. Interestingly, when grouping the cases according to whether they contained additional chromosomal aberrations (ACA) or not there is tendency that T/NT nuclei with no ACA (Cases 1, 3 and 5) only have a limited increase in the contour area compared with T/NT nuclei without ACA (Cases 2, 4, 6 and 7) (Figure 3C). This phenomenon has not been described before because it is not possible to compare measures of cell size with respect to ploidy and presence or absence of ACA by morphological examination alone. Blast morphology is the association most frequently cited with T/NT AML and provides a morphological clue to the underlying karyotype as it is generally believed that increased DNA content is directly related to blast size [23,24]. These results indicate that if giant cells are found in BM aspirates the presence of T/NT karyotypes should also be considered [3,25]. However, the present data indicate that in some cases the T/NT nucleus is not enlarged (Case 1) or enlarged only to a very limited degree (−ACA compared with +ACA) and is thereby at risk of being missed by morphological examination alone. Only few studies exist, and it is not clear how cell volume is regulated by the ploidy level [26]. Further studies are awaited to clarify this but as cases with tetraploid karyotypes are rare this may take a while.

**Figure 4:**
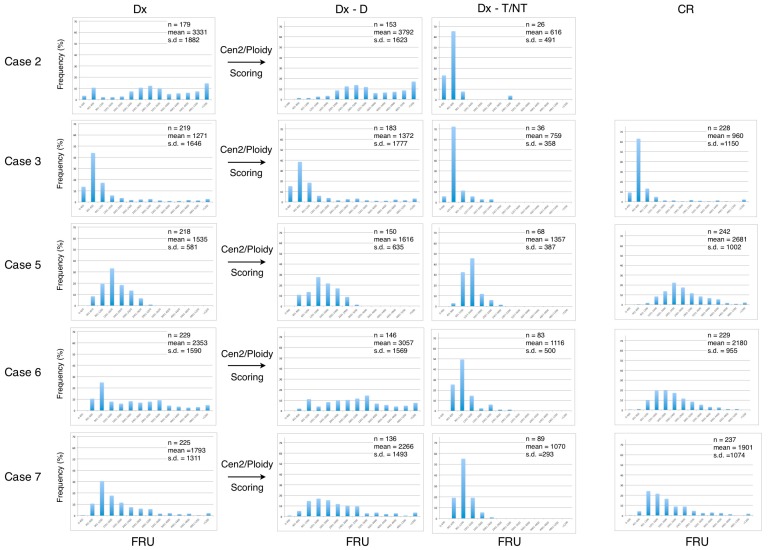
Quantification of telomere content in bone marrow cells at diagnosis (Dx) and at complete remission (CR) in the AML disease group. The FRU values were grouped and frequencies were normalized at Dx, and after ploidy scoring into Dx-Diploid and Dx-T/NT nuclei as well as at CR. At CR there were no clonal T/NT cells present in either of the samples. In the upper right corner of each histogram is indicated: the number of analyzed cells (n), the mean values, and standard deviation (SD).

**Figure 5:**
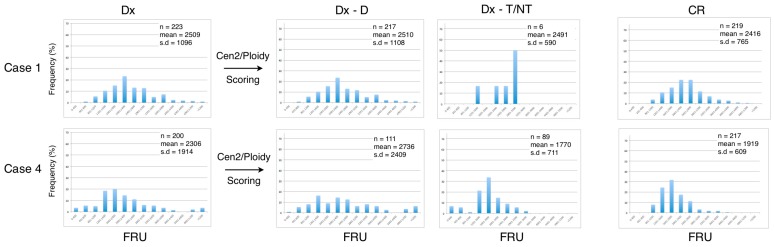
Quantification of telomere content in bone marrow cells at diagnosis (Dx) and at complete remission (CR) in the B-cell disease group. The FRU values were grouped and frequencies were normalized at Dx, and after ploidy scoring into Dx-Diploid and Dx-T/NT nuclei as well as at CR as indicated in Figure 4.

We next examined the dataset of the telomere measurements from each of the seven included patients at diagnosis (Dx), after manually ploidy scoring of analyzed cells (Dx-D and Dx-T/NT) and at CR (Figure 4 and Figure 5). The frequency distribution of grouped telomere content in the AML disease group shows that the profiles from the Dx-T/NT nuclei are skewed toward lower telomere content compared with Dx-D nuclei, and that the Dx-D nuclei in general have a greater variance (Figure 4). When the AML patients obtained CR there was a tendency for the telomere content to increase and for the distribution to become Gaussian. The frequency distribution of telomere content in the B-cell disease group shows that there are no major differences between telomere content at diagnosis and at CR and that the profiles are compatible with higher telomere content in Dx-T/NT compared with Dx-T/NT in the AML disease group (Figure 5).

**Figure 6:**
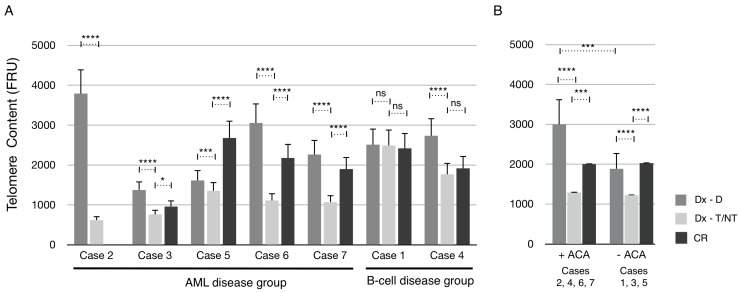
Mean telomere content of patients in the AML and B-cell disease groups. (**A**) Telomere content in the nuclei of each patient at diagnosis and after ploidy scoring into diploid (Dx-D) or as T/NT (Dx-T/NT) nuclei, and at CR. (**B**) Average telomere content when nuclei were grouped according to presence of additional chromosomal aberrations (+ACA) or their absence (−ACA) after ploidy scoring into diploid (Dx-D) or T/NT (Dx-T/NT) nuclei, and at CR. After significance testing number of asterisk indicates level of significance as described in Figure 2.

The mean telomere content of all seven cases at diagnosis (Dx) after manual scoring of ploidy (Dx-D and Dx–T/NT) and at CR was determined and plotted (Figure 6A). In the AML disease group, the mean telomere content was significantly reduced in Dx-T/NT nuclei compared with Dx-D nuclei in all cases. Furthermore, all cases had a significant increase in mean telomere content at CR compared with Dx-T/NT. However, only in one case (Case 5) the mean telomere content was increased above the level at Dx-D nuclei. In the B-cell disease group the telomere content was unchanged in Case 1, and in Case 4 there was a significant reduction in telomere content in Dx-T/NT nuclei compared with Dx-D nuclei. Interestingly, when grouping the cases according to whether the T/NT nuclei contained ACA or not it is clear that there are no significant differences in the mean telomere content between these two groups (Figure 6B). However, the mean telomere content in the +ACA group was reduced to a greater extent compared with the –ACA group. Furthermore, at CR there was an increase in telomere content to a similar extent as diploid nuclei at diagnosis compared with T/NT nuclei in both ACA groups. Together these results indicate that T/NT nuclei have a significant reduction in mean telomere content that can be restored at CR.

## 4. Conclusions and Comments

In agreement with previous studies [13] we have found that T/NT karyotypes are very rare in hematological malignancies. We identified seven patients with T/NT karyotypes in the five-year period from June 2012 to May 2017 out of 12,000 examined adult cases in this period.

We used the iQFISH method, which is a single cell-based method for telomere length analysis that allows for telomere measurements in different cell types according to ploidy without having to sort cells in advance. Furthermore, it is an automated analysis on interphase nuclei and does not require mitotic cells for analysis. We showed a highly significant shortening in average telomere length in T/NT nuclei compared with diploid nuclei. The telomere shortening is more pronounced in the AML disease group compared with the B-cell disease group. Further, we find that the telomere content, in general, is restored at CR. There may be several explanations for this interesting observation, all of which, however, require additional studies.

The average telomere content in T/NT nuclei with ACA and without ACA is significantly reduced to a similar level in the two groups compared with diploid cells. Furthermore, our data suggest that low telomere content is associated with T/NT cells, whereas presence of ACA is not associated with additional decrease in telomere content.

A limitation of many telomere studies is that it is the average leukemic cell telomere length that is studied and most often T/NT and diploid cells are mixed populations. We find that it is important to distinguish between cell types according to ploidy to enhance the possibility of identifying possible differences in telomere content between nuclei with T/NT and diploid chromosomal complement. The iQFISH method described here has the advantage that telomere measurements, ploidy level, and nucleus size can be evaluated in single cells in a single experiment.

Previous studies showed that tetraploid AML often displays large and bizarre blast cells [3]. Here we showed that the nucleus size in T/NT nuclei is enlarged by a factor of more than 4 but also that there is great variability between patients. Interestingly, we found that the nucleus size is only enlarged to a limited degree in cells without ACA compared with cells with ACA.

In agreement with previous studies, we have found that leukemia with T/NT has a high degree of malignancy and patient survival is relatively short [27]. It is therefore of great importance to elucidate the mechanisms of tetraploidization in hematological malignancies and its role in CIN.

## Figures and Tables

**Table 1:** Summary of cytogenetic findings in patients with tetraploid/near-tetraploid (T/NT) karyotypes.

Case	Age/Gender	Diagnosis	Karyotype at Diagnosis	Additional Chromosomal Aberrations	Remission Karyotype (Month after Diagmosis)	Outcome
1	49/F	B-ALL	92,XXXX[7]/46,XX[18].nuc ish (MYCx4)[9/200]	No	46,XX[25] (1 mo.)	Alive, >5 years
2	82/M	AML with intestinal sarcoma	91–94,XXYY,i(1)(q10),i(2)(q10),+8,−14,+16 [cp 7]/46,XY[18].nuc ish (MYCx4) [30/200]	Yes	No remission sample	Died 32 days after diagnosis
3	67/M	sAML from MDS	92,XXYY[3]/46,XY[22].nuc ish (PDGFRAx4)[66/200],(PDGFRBx4)[74/200], (FGFR1x4)[66/200],(BCRx4)[70/200]	No	46,XY[25] (4 mo.)	Died 252 days after diagnosis
4	70/M	Follicular lymphoma	82−86,XYY,der(X)t(X;3)(q11;p or q),−2, der(3)t(3;10)(p11;p or q),−4,−4,−6, ins(7;13)(q11;q?q?)x2,−9,−10, der(11)t(4;11)(p or q;p12),der(13)t(7;13)(p or q;q14),t(14;18)(q32;q21)x2, −15, ins(15;17)(q22;?q12q25), der(16)t(2;16)(q2?4;q2?2),del(17)(q12), der(21;21)(q10;q10),−22[cp 19]/46,XY[6].nuc ish (MYCx4)[103/200]	Yes	46,XY[25] (4 mo.)	Died 296 days after diagnosis
5	73/F	AML (M2)	92,XXXX[19]/46,XX[6].nuc ish (CBFBx4)[99/200]	No	46,XX[25] (2 mo.)	Died 695 days after diagnosis
6	64/F	AML (M1/M2)	89−92,XXXX,inc[cp 7]/46,XX[18].nuc ish (CBFBx4)[95/200]	Yes	46,XX[25] (1 mo.)	Alive at 687 days after HSCT was done (which was 140 days after diagnosis)
7	75/M	AML	92,XXY,−Y,del(2)(q13q24),+13,+15,−21,−22[cp 12]/45,X,−Y[5]/46,XY[8].nuc ish (CBFBx4)[128/200], (RARAx4)[119/200]	Yes	46,XY[25] (1 mo.)	Alive at 168 days after diagnosis

HCST: Hematopoietic stem cell transplantation; F: female; M: male; B-ALL: B-cell acute lymphoblastic leukemia; AML: acute myeloid leukemia; sAML: secondary AML; MDS: myelodysplastic syndrome; mo.: months.
